# Phosphorylation of Histone H2A.X in Peripheral Blood Mononuclear Cells May Be a Useful Marker for Monitoring Cardiometabolic Risk in Nondiabetic Individuals

**DOI:** 10.1155/2017/2050194

**Published:** 2017-05-09

**Authors:** So Ra Yoon, Juhyun Song, Jong Hwa Lee, Oh Yoen Kim

**Affiliations:** ^1^Department of Food Science and Nutrition, Brain Busan 21 Project, Dong-A University, Busan 49315, Republic of Korea; ^2^Department of Biomedical Sciences, Center for Creative Biomedical Scientists, Chonnam National University, Gwangju 61469, Republic of Korea; ^3^Department of Rehabilitation Medicine, Dong-A University Hospital, Busan 49315, Republic of Korea; ^4^Human Life Research Center, Dong-A University, Busan 49315, Republic of Korea

## Abstract

Phosphorylation of H2A.X (serine 139) in the histone H2A family located in the downstream of the DNA damage kinase signaling cascade is an important indicator of DNA damage. Recently, phosphorylation of H2A.X was proposed as a sensitive biomarker of aging. This study investigated if phosphorylation of H2A.X in peripheral blood mononuclear cells (PBMCs) is associated with cardiometabolic risk in nondiabetic individuals. Basic parameters and oxidative stress/inflammatory markers were measured in nondiabetic healthy Koreans (*n* = 119). Phosphorylation of H2A.X was measured randomly among the study subjects using a flow cytometer. According to the number of metabolic syndrome risk factor (MetS-RF), the study subjects were subdivided into “super healthy” (MetS − RF = 0, *n* = 71) and “MetS-risk” (MetS − RF ≥ 1, *n* = 48) groups. Phosphorylation of H2A.X in PBMCs (percentages and mean fluorescence intensity) was significantly higher in the MetS-risk group than in the super healthy group after adjusting for age, sex, cigarette smoking, and alcohol consumption. Phosphorylated H2A.X was positively correlated with the number of MetS-RF as well as waist circumference, blood pressures, triglyceride, Hb_A1C_, oxidized LDL, high sensitivity C-reactive protein, tumor necrosis factor-alpha, and alanine aminotransferase after the adjustment. The present study suggested that phosphorylated H2A.X in circulating PBMCs measured by flow cytometer may be a useful marker for monitoring cardiometabolic risk in nondiabetic individuals.

## 1. Introduction

Cardiometabolic syndrome is one of the major public health problems in the world, which develops to type 2 diabetes mellitus (T2DM) and cardiovascular disease (CVD) [1]. Major risk factors of cardiometabolic syndrome include overweight/obesity, metabolic syndrome (MetS), hypertension, dyslipidemia, or glycemic disorder (i.e., impaired glucose and insulin resistance) [2]. Li et al. reported the association between cardiometabolic risk factors and changes for cardiac structure/function in patients with early-stage heart failure [3]. Previous studies have demonstrated that patients with diabetes had sevenfold higher risk of CVD than nondiabetic individuals [4, 5], and people with impaired glucose tolerance had almost twice higher risk of developing CVD than those with normal blood glucose levels [6].

Impaired balance between oxidative stress (i.e., reactive oxygen species (ROS)) and antioxidants is considered as one of the primary causes in the pathogenesis of chronic diseases such as diabetes and CVD and their complications [7–12]. Low levels of ROS produced during the metabolic processes promote cell growth and participate in stress adaptation, injury responses, and various modifications in the cellular phenotype [7]. However, excessively high levels of ROS induce cellular apoptosis and tissue injury and trigger oxidative stress and inflammatory response, thereby impairing cellular functions and inducing various metabolic dysfunctions [7, 13, 14].

Higher oxidative stress is involved in the pathogenesis of oxidative damage in cellular proteins, membrane lipids, and DNA [15]. Double-strand breaks (DSBs) created in the eukaryotes are generally accompanied by the formation of hundreds of histone H2A.X molecules in the chromatin flanking the DSBs [16]. H2A.X is a member of the histone H2A family which resides downstream of the DNA damage kinase signaling cascade [17, 18]. Phosphorylation of histone H2A.X on serine 139 which is also known as *γ*-H2A.X is an important indicator of DNA damage [19]. Recent studies suggested that H2AX and its phosphorylation on serine 139, beyond their role as a marker for DNA damage, may be a sensitive molecular biomarker of aging and related disease which is accumulated in senescent human cells [20, 21]. However, there is no study on the relationship between phosphorylation of H2A.X in circulating PBMCs and the risk of metabolic disease in healthy people. Therefore, this study investigated if phosphorylation of H2A.X in PBMCs is associated with oxidative stress, and inflammatory response, and reflects cardiometabolic risk in nondiabetic individuals.

## 2. Methods

### 2.1. Study Participant and Design

Study participants were recruited from the Health Promotion Center at Dong-A University Hospital between January and March 2014. Subjects were excluded if they had orthopedic limitations or had ≥10% of weight loss/gain over the previous 6 months or any diagnosis or history of chronic diseases (e.g., diabetes, vascular disease, heart disease, renal disease, and liver disease), cancer (clinically or by anamnesis), pregnancy, breast feeding, or intending to become pregnant during the time of this study. All the participants were provided with detailed information of the study and provided written informed consent. The participants were interviewed to determine their smoking and drinking behavior. The study protocol was approved by the Institutional Review Board of Dong-A University and was carried out in accordance with the Declaration of Helsinki. Finally, 119 individuals were included in the study and subdivided into two groups: “super healthy” people (MetS risk factor = 0, *n* = 71) and “MetS risk” carriers (MetS risk factor ≥ 1, *n* = 48) based on MetS risk factors (waist circumference ≥ 90 cm for male and ≥80 cm for female; systolic blood pressure ≥ 130 mmHg or diastolic blood pressure ≥ 85 mmHg; fasting glucose ≥ 100 mg/dL; fasting triglycerides ≥ 150 mg/dL; HDL cholesterol < 40 mg/dL for male and <50 for female) [22–26].

### 2.2. Anthropometric Parameter, Blood Pressure, and Blood Collection

Body mass index (BMI) was calculated as body weight divided by height (kg/m^2^). Waist circumference was measured at the umbilicus with the subject standing. Blood pressure (BP) was obtained from the left arm of the seated individuals with an automatic BP monitor (HEM-7220, Omron, Matsusaka, Japan) after getting a short rest. After a 12 h fast, venous blood specimens were collected in EDTA-treated and plain tubes, separated to plasma or serum, and stored at −80°C until analysis. Blood specimens for flow cytometric analysis of phosphorylated H2A.X expression in circulating peripheral blood mononuclear cells (PBMCs) were collected from the participants who agreed to the measurement.

### 2.3. Serum Lipid Profile, Fasting Glucose, and Glycated Hemoglobin (Hb_A1C_)

Fasting total cholesterol and triglycerides were analyzed by enzymatic assays using commercially available kits on a Hitachi 7150 Autoanalyzer (Hitachi Ltd., Tokyo, Japan). After precipitation of chylomicrons with dextran sulfate magnesium, levels of low-density lipoprotein (LDL) and high-density lipoprotein (HDL) cholesterol in the supernatants were measured by an enzymatic method. Fasting glucose levels were determined by the glucose oxidase method with Beckman Glucose Analyzer (Beckman Instruments, Irvine, CA, USA). Glycated hemoglobin (Hb_A1C_) was measured using VARIANT II Turbo Hb_A1C_ kit-2.0 (Bio-Rad, Hercules, CA, USA).

### 2.4. Oxidative Stress and Inflammation Markers

Plasma oxidized LDL (oxLDL) was measured using an enzyme immunoassay (Mercodia, Uppsala, Sweden). The resulting color reaction was measured using the iMark™ microplate absorbance reader (Bio-Rad Laboratories, Hercules, CA, USA). The wavelength correction was set to 450 nm and 540 nm. Serum high-sensitivity C-reactive protein (hs-CRP) was measured with an ADVIA 1650 system (Bayer, Tarrytown, NY, USA) using a commercially available, high-sensitivity CRP-Latex(II) X2 kit (Seiken Laboratories Ltd., Tokyo, Japan). Plasma tumor necrosis factor-*α* (TNF-*α*) was measured using human Quantikine HS ELISA Kit (R&D system, Minneapolis, MN USA). The resulting color reaction was measured using the iMark microplate absorbance reader (Bio-Rad Laboratories, Hercules, CA, USA). The wavelength correction was set to 490 nm and 560 nm. White blood cell counts were determined using the HORIBA ABX diagnostic (HORIBA ABX SAS, Parc Euromedicine, France).

### 2.5. Liver and Kidney Function Parameters

Serum levels of aspartate aminotransferase (AST) and alanine aminotransferase (ALT) were measured by a kinetic UV method based on recommendations by the International Federation of Clinical Chemistry using commercially available kits on a Hitachi 7180 Autoanalyzer (Hitachi Ltd., Tokyo, Japan). Serum levels of blood urea nitrogen (BUN) and creatinine were measured by a kinetic UV assay (Hitachi Ltd., Tokyo, Japan). Serum creatinine was measured by a kinetic colorimetric assay (Jaffe).

### 2.6. Phosphorylation of Histone H2A.X in Peripheral Blood Mononuclear Cells

Blood specimens for flow cytometric analysis of phosphorylated H2A.X expression in circulating peripheral blood mononuclear cells (PBMCs) were collected from the participants who agree to the measurement as mentioned above. Whole blood was mixed with the same volume of RPMI 1640 medium (HyClone, Logan, UT, USA) and gently laid on a histopaque-1077 (Sigma-Aldrich, St. Louis, MO, USA). The sample was then centrifuged at 1800 rpm for 20 min at 10°C. After the separation, a thin layer of PBMCs was isolated and washed twice with RPMI 1640. The pellet was resuspended in RPMI 1640 with streptomycin [27, 28]. Phosphorylation of histone H2A.X in PBMCs was measured with the Muse™ H2A.X activation dual detection kit (MCH200101, Millipore, Billerica, MA, USA). Briefly, isolated PBMCs were counted and diluted with assay buffer (5 × 10^6^ cells/2.5 ml). After that, the cells were fixed, permeabilized, and incubated with antiphosphohistone H2A.X (Ser139, Alexa Fluor®555, part number CS208203, Millipore, Billerica, MA, USA) and anti-H2A.X (PECy5, part number CS209202, Millipore, Billerica, MA, USA), and then analyzed by the Muse cell analyzer (Millipore, Billerica, MA, USA) following the manufacturer's protocol. Each sample for the phosphorylated H2A.X measurement was prepared in triplicates; then, the average values were used in the statistical analysis.

### 2.7. Statistical Analysis

Statistical analyses were performed using SPSS ver22.0 for Windows (SPSS Inc., Chicago, IL, USA). The Student *t*-test was used to compare parameters between the two groups. A general linear model analysis followed by Bonferroni correction was also performed to evaluate the differences in the parameters between the groups after adjustment for the confounding factors (i.e., age, sex, cigarette smoking, and alcohol consumption). Frequency was tested with the chi-square test. The relationships between phosphorylated H2A.X and cardiometabolic risk parameters were tested by *partial* correlation analyses after adjusting for confounding factors. The distribution of continuous variables was inspected to detect nonnormal distribution before statistical analysis. Skewed variables were log-transformed for statistical analysis (i.e., fasting glucose, triglyceride, oxidized LDL, TNF-*α*, and phosphorylated H2A.X parameters (percentage and MFI)). For descriptive purposes, the mean values are presented using untransformed values (expressed as means ± standard error or percentages). A two-tailed *p* value of less than 0.05 was considered statistically significant.

## 3. Results

### 3.1. General Characteristics and Cardiometabolic Risk Parameters of the Study Subjects

Table 1 shows the general characteristics and MetS risk-related biochemical parameters of the study subjects. MetS risk carriers were older, showed higher proportion of men, and consumed more cigarette and alcohol than super healthy people. After adjusting for age, sex, cigarette smoking, and alcohol consumption, the MetS risk group showed higher levels of BMI, waist circumference, blood pressures, fasting glucose, Hb_A1C_, triglyceride, LDL cholesterol, and total cholesterol and lower levels of HDL cholesterol than the super healthy group (*p* < 0.05 for all) even though the average values of the parameters in both groups were in normal range. In addition, the levels of oxidative stress (oxidized LDL), inflammation (hs-CRP, TNF-*α*, white blood cell counts), and liver (ALT) and kidney (BUN) function markers were significantly higher in the MetS risk group than those in the super healthy group (*p* < 0.05 for all) (Table 2).

### 3.2. Phosphorylated H2A.X in Peripheral Blood Mononuclear Cells between Super Healthy People and the MetS Risk Carriers

**Figure 1:**
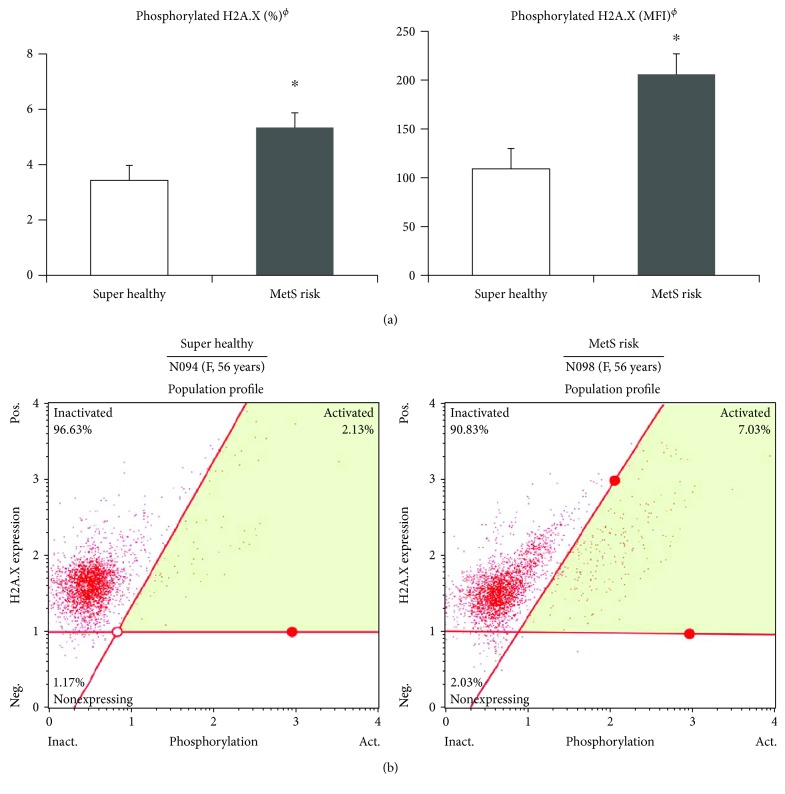
Phosphorylated H2A.X (percentage, MFI) between the super healthy and MetS risk groups. (a) Phosphorylated H2A.X (percentage and MFI) levels expressed in peripheral blood mononuclear cells (PBMCs) between the super healthy group (*n* = 18) and the MetS risk group (*n* = 17). Data are means ± S.E. ^ϕ^Tested after log-transformed, ^∗^*p* < 0.05; tested by general linear model method adjusted for age, sex, cigarette smoking, and alcohol consumption. (b) Representative flow cytometric analysis images for phosphorylation of H2A.X expressed on PBMCs in age- and sex-matched individuals (56 years, females: super healthy: *n* = 1; MetS risk: *n* = 1). MetS: metabolic syndrome; MFI: mean fluorescence intensity.

As mentioned in Section 2, phosphorylations of H2A.X expressed in circulating PBMCs were randomly measured among the study subjects who agree to the measurement. After the adjustment for age, sex, cigarette smoking and alcohol consumption, and percentage and mean fluorescence intensity (MFI) of phosphorylated H2AX were still significantly higher in the MetS risk carriers (*n* = 17) than in super healthy people (*n* = 18) (for percentage, super healthy: 3.44 ± 0.53% versus MetS risk: 5.34 ± 0.70%; for MFI, super healthy: 109.1 ± 21.1 versus MetS risk: 205.9 ± 39.3) (*p* < 0.05 for all) (Figure 1(a)). In addition, Figure 1(b) represents the images of flow cytometric analysis for phosphorylation of H2A.X expressed in PBMCs from super healthy (*n* = 1) and MetS risk (*n* = 1) individuals who were age and sex matched (56 years, females).

### 3.3. Relationships between Phosphorylation of H2A.X in PBMCs and Cardiometabolic Risk Parameters

**Figure 2:**
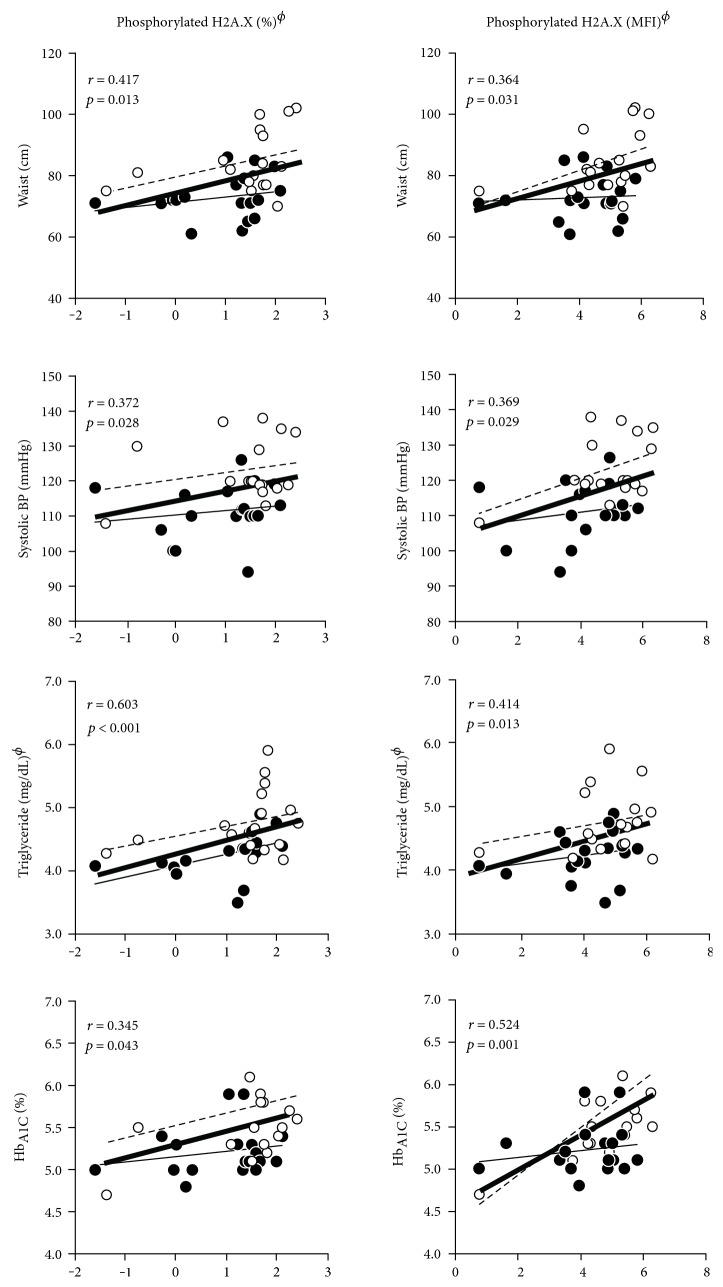
Relationships between phosphorylated H2A.X (percentage, MFI) and MetS risk related parameters. *r*: correlation coefficient, tested by *partial* correlation analysis adjusted for age, sex, cigarette smoking, and alcohol consumption; ^ϕ^tested after log-transformed; super healthy (•, **—:** narrow solid line) (MetS risk factor = 0) and MetS risk (o, - - -: narrow open line) (MetS risk factor ≥ 1) groups and total subjects (**—**: bold solid line); BP: blood pressure; Hgb_A1C_: hemoglobin A1C; MFI: mean fluorescence intensity.

**Figure 3:**
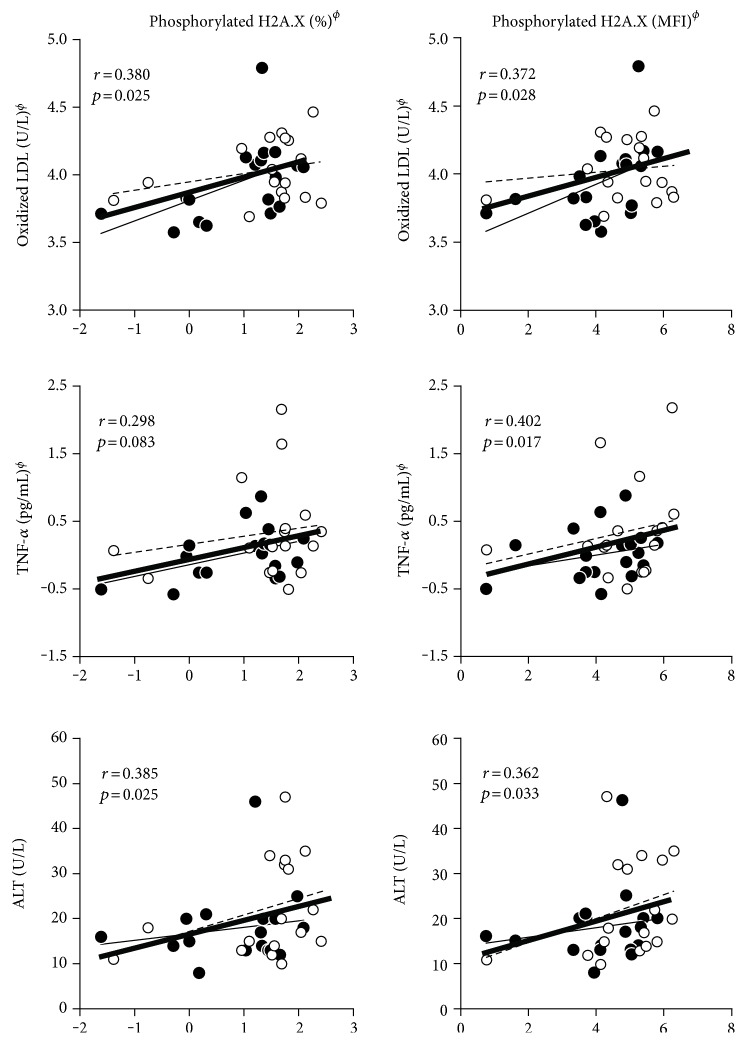
Relationships between phosphorylated H2A.X (percentage, MFI) and oxidative stress and inflammation and liver function markers. *r*: correlation coefficient, tested by *partial* correlation analysis adjusted for age, sex, cigarette smoking, and alcohol consumption; ^ϕ^tested after log-transformed; super healthy (•, **—**: narrow solid line) (MetS risk factor = 0) and MetS risk (o, - - -: narrow open line) (MetS risk factor ≥ 1) groups and total subjects (**—**: bold solid line). ALT: alanine aminotransferase; hs-CRP: high-sensitivity C-reactive protein; MFI: mean fluorescence intensity; TNF-*α*: tumor necrosis factor-alpha.

Phosphorylated H2A.X in PBMCs was positively correlated with age (for percentage: *r* = 0.526, *p* = 0.001; for MFI: *r* = 0.609, *p* < 0.0001). Therefore, correlation analysis between phosphorylated H2A.X and cardiometabolic risk parameters was performed with adjustment for age, sex, cigarette smoking, and alcohol consumption. Figures 2() and 3() present the relationship between phosphorylated H2A.X (both percentage and MFI) and each of the MetS risk components and the related parameters. Precisely, correlation dot plots were separately presented for the super healthy group (MetS risk factor = 0) and the MetS risk group (MetS risk factor ≥ 1). As shown in Figure 2(), both percentage and MFI of the phosphorylated H2A.X were positively correlated with each of MetS risk components in both subject groups, particularly in the MetS risk group: positively correlated with waist circumference (*r* = 0.417, *p* = 0.013; *r* = 0.364, *p* = 0.031, resp.), systolic BP (*r* = 0.372, *p* = 0.028; *r* = 0.369, *p* = 0.029, resp.), triglycerides (*r* = 0.603, *p* < 0.001; *r* = 0.414, *p* = 0.013, resp.), and Hb_A1C_ (*r* = 0.345, *p* = 0.043; *r* = 0.524, *p* = 0.001, resp.).  Figure 3() also presents that both percentage and MFI of phosphorylated H2A.X were positively correlated with oxidized LDL (*r* = 0.380, *p* = 0.025; *r* = 0.372, *p* = 0.028, resp.) and ALT (*r* = 0.385, *p* = 0.025; *r* = 0.362, *p* = 0.033, resp.). TNF-*α* levels were also positively correlated with MFI of phosphorylated H2A.X (*r* = 0.402, *p* < 0.017), but the relationship between TNF-*α* levels and percentage of phosphorylated H2A.X turned to be a tendency (*r* = 0.298, *p* < 0.083). Phosphorylated H2A.X was also positively correlated with the number of MetS risk factor (for percentage: *r* = 0.338, *p* = 0.047; for MFI: *r* = 0.320, *p* = 0.061, resp.). In addition, two phosphorylated H2A.X parameters were compared according to the level of each MetS component (see Supplementary Figure S1 available online at https://doi.org/10.1155/2017/2050194). Interestingly, some of the MetS risk factors (i.e., particularly, waist circumference, triglyceride, Hb_A1C_, and blood pressure) were distinctly associated with phosphorylated H2A.X parameters: subjects with higher waist circumference showed significantly higher values of phosphorylated H2A.X parameters (both percentage and MFI), those who had higher fasting triglyceride also showed higher percentage of phosphorylated H2A.X, and those who had higher Hb_A1C_ showed higher MIF of phosphorylated H2A.X. Regarding BPs, subjects with higher BPs showed increased tendency of higher MFI of phosphorylated H2A.X.

## 4. Discussion

The present study shows that phosphorylation of histone H2A.X in circulating PBMCs was significantly higher in the MetS risk group than in the super healthy group. In addition, phosphorylated H2A.X was positively correlated with the number of MetS risk factors as well as waist circumference, triglyceride, Hb_A1C_, blood pressures, oxidized LDL, inflammatory markers (i.e., hs-CRP and TNF-*α*), and ALT. The statistical significances were maintained after adjusting for age, sex, cigarette smoking, and alcohol consumption. Flow cytometric analysis of phosphorylation of H2A.X in circulating PBMCs was used for the first time for monitoring cardiometabolic risk in nondiabetic individuals.

Oxidative stress is a primary cause of deleterious oxygen free radical formed through cytosolic NADPH oxidases and impaired balance of the antioxidant system [7]. These abnormal reactions cause DNA damage and generate phosphorylation of the histone variant H2A.X that is localized to DSB signaling response [29, 30]. Phosphorylated H2A.X may facilitate an appropriate awareness of DNA damage by oxidative stress and/or inflammatory responses and also may be a predictor for the increased risk of CVD and various metabolic disorders [31, 32]. Demirbag et al. indicated that the extent of DNA damage is much higher in patients with MetS than in those without MetS [33]. An in vitro study shows that phosphorylation of H2A.X (*γ*-H2A.X) in human endothelial cells is more upregulated under stably high glucose status, particularly under oscillating glucose status than under normal glucose status [34]. Accumulation of DNA damage such as *γ*-H2A.X and DSB formation by oxygen free radical is a typical theory of aging along with telomere length [7]. Recently, Schurman et al. suggested that *γ*-H2A.X may be a biomarker for human morbidity in age-related diseases [21]: the number of *γ*-H2A.X foci in human blood cells was higher in the middle-aged subjects (50–59 years) than in the younger subjects (35–49 years) and the number of *γ*-H2AX foci/cell in patients with hypertension was 36% higher than those in nonhypertensive patients, particularly among those ≥57 years [21]. In addition, Scarpato et al. reported that nuclear damages expressed by H2A.X foci and micronucleus in peripheral lymphocytes were at least 5 times higher in obese/overweight children than in normal weight children, and they are related to higher inflammation status (i.e., TNF-*α*, IL-6, and CRP) [35]. These reports are partly in accordance with our results. In our study, phosphorylated H2A.X was positively correlated with the number of MetS risk factors as well as waist circumference, systolic BP, triglyceride, Hb_A1C_, oxidized LDL, hs-CRP, and TNF-*α* which are considered as cardiometabolic risk factors. These significances were maintained after adjusting for confounding factors (i.e., age, sex, cigarette smoking, and alcohol consumption).

In addition, epidemiological and clinical studies estimated that liver enzymes might be useful biomarkers for MetS and other lifestyle-related diseases, even though the enzymes are within the normal range [36–39]. For example, Zhang et al. shows the positive associations between MetS risk and continuous unit (per 5 unit) of liver enzyme (ALT, AST, *γ*-glutamyl transpeptidase, and alkaline phosphatase): the smallest effect size was for alkaline phosphatase [odds ratio (OR): 1.09, 95% confidence interval (CI): 1.08–1.10] and the largest was for ALT (OR: 1.41, 95% CI: 1.38–1.43). Particularly, subjects with relatively higher ALT levels (>27 U/L) showed higher OR for MetS risk (8.03, CI: 7.06–9.12) than those with lower ALT levels (<15 U/L) among the ALT quartile groups after adjusting for confounding factors (i.e., age, sex, and education level) [36]. In our study, ALT levels were significantly higher in MetS risk carriers than in super healthy people although the average values are within the normal range [39] and positively correlated with phosphorylation of H2A.X in circulating PBMCs after the adjustment.

However, our study may have limitations. First, the study design was based on cross-sectional observation, not on a case-control design, because the study subjects were classified by screening their anthropometric and metabolic values on the day of participation even though some people in the borderline of criteria had re-examination. Second, the study population is relatively small, although the statistical significances were maintained even after the adjustment for confounding factors (age, sex, cigarette smoking, and alcohol consumption). Therefore, a further study with a large number of subjects especially for measuring H2A.X phosphorylation using flow cytometer should be performed to verify our results. Despite the study limitations, the present study suggested that phosphorylated H2A.X in circulating PBMCs measured by flow cytometer may be a useful marker for monitoring cardiometabolic risk in nondiabetic individuals.

## Supplementary Material

Some of the MetS risk factors (i.e., particularly, waist circumference, triglyceride, HbA1C, and blood pressure) were distinctly associated with phosphorylated H2A.X parameters: subjects with higher waist circumference showed significantly higher values of phosphorylated H2A.X parameters (both percentage and MFI), those who had higher fasting triglyceride also showed higher percentage of phosphorylated H2A.X, and those who had higher HbA1C showed higher MIF of phosphorylated H2A.X. Regarding BPs, subjects with higher BPs showed increased tendency of higher MFI of phosphorylated H2A.X.

## Figures and Tables

**Table 1:** General characteristics and metabolic risk-related biochemical parameters of the study subjects.

	Super healthy (*n* = 71)	MetS risk (*n* = 48)
Age (year)	45.9 ± 1.24	54.9 ± 1.56^+^
Male, *n* (%)	7 (10.0)	17 (35.4)^+^
Cigarette (*n*/day)	1.34 ± 0.57	5.73 ± 2.00^+^
Alcohol (g/week)	20.8 ± 2.80	38.0 ± 10.9^+^
BMI (kg/m^2^)	21.9 ± 0.27	25.9 ± 0.48^∗^
Waist (cm)	72.4 ± 0.77	84.8 ± 1.47^∗^
Systolic BP (mmHg)	109.8 ± 0.99	120.3 ± 1.74^∗^
Diastolic BP (mmHg)	69.7 ± 0.70	74.1 ± 1.07^∗^
Glucose (mg/dL)^ϕ^	85.4 ± 0.82	100.1 ± 2.21^∗^
Hb_A1C_ (%)	5.17 ± 0.04	5.65 ± 0.08^∗^
Triglyceride (mg/dL)^ϕ^	67.6 ± 3.48	144.7 ± 11.9^∗^
HDL cholesterol (mg/dL)	69.5 ± 1.50	52.9 ± 1.97^∗^
LDL cholesterol (mg/dL)	115.0 ± 3.05	132.6 ± 5.30^∗^
Total cholesterol (mg/dL)	187.7 ± 2.92	202.0 ± 6.13^∗^

Data are means ± S.E. or percentage (%); ^ϕ^tested after log-transformed, ^+^*p* < 0.05; tested by Student's *t*-test or chi-square method, ^∗^*p* < 0.05; tested by general linear model method adjusted for age sex, cigarette smoking, and alcohol consumption. AST: aspartate aminotransferase; ALT: alanine aminotransferase; BMI: body mass index; BP: blood pressure; BUN: blood urea nitrogen; Hb_A1C_: hemoglobin A1C.

**Table 2:** Oxidative stress, inflammation, and liver and kidney function markers between super healthy people and MetS risk carriers.

	Super healthy (*n* = 71)	MetS risk (*n* = 48)
Oxidized LDL (U/L)^ϕ^	54.9 ± 2.08	62.2 ± 3.17^∗^
hs-CRP (mg/dL)^ϕ^	0.51 ± 0.17	0.84 ± 0.25^∗^
TNF-*α* (pg/mL)^ϕ^	1.07 ± 0.07	1.69 ± 0.30^∗^
White blood cell (×10^9^/L)	4.92 ± 0.15	5.65 ± 0.21^∗^
AST (U/L)	25.0 ± 0.88	27.2 ± 1.31
ALT (U/L)	20.0 ± 1.37	27.3 ± 2.14^∗^
Creatinine (mg/dL)	0.77 ± 0.01	0.87 ± 0.07
BUN (mg/dL)	13.2 ± 0.38	14.2 ± 0.73^∗^

Data are means ± S.E.^ϕ^Tested after log-transformed, ^∗^*p* < 0.05; tested by general linear model method adjusted for age, sex, cigarette smoking, and alcohol consumption. hs-CRP: high-sensitivity C-reactive protein; TNF-*α*: tumor necrosis factor-alpha.
