# Supplementary material for: Phosphorylation of Histone H2A.X in Peripheral Blood Mononuclear Cells May Be a Useful Marker for Monitoring Cardiometabolic Risk in Nondiabetic Individuals
Source: Dis Markers. 2017 May 9;2017:2050194. doi: 10.1155/2017/2050194 (PMC5441116; doi:10.1155/2017/2050194)
Supplement: Supplementary file 1 — Some of the MetS risk factors (i.e., particularly, waist circumference, triglyceride, HbA1C, and blood pressure) were distinctly associated with phosphorylated H2A.X parameters: subjects with higher waist circumference showed significantly higher values of phosphorylated H2A.X parameters (both percentage and MFI), those who had higher fasting triglyceride also showed higher percentage of phosphorylated H2A.X, and those who had higher HbA1C showed higher MIF of phosphorylated H2A.X. Regarding BPs, subjects with higher BPs showed increased tendency of higher MFI of phosphorylated H2A.X. [file 2050194.f1.pptx]

## Slide 1
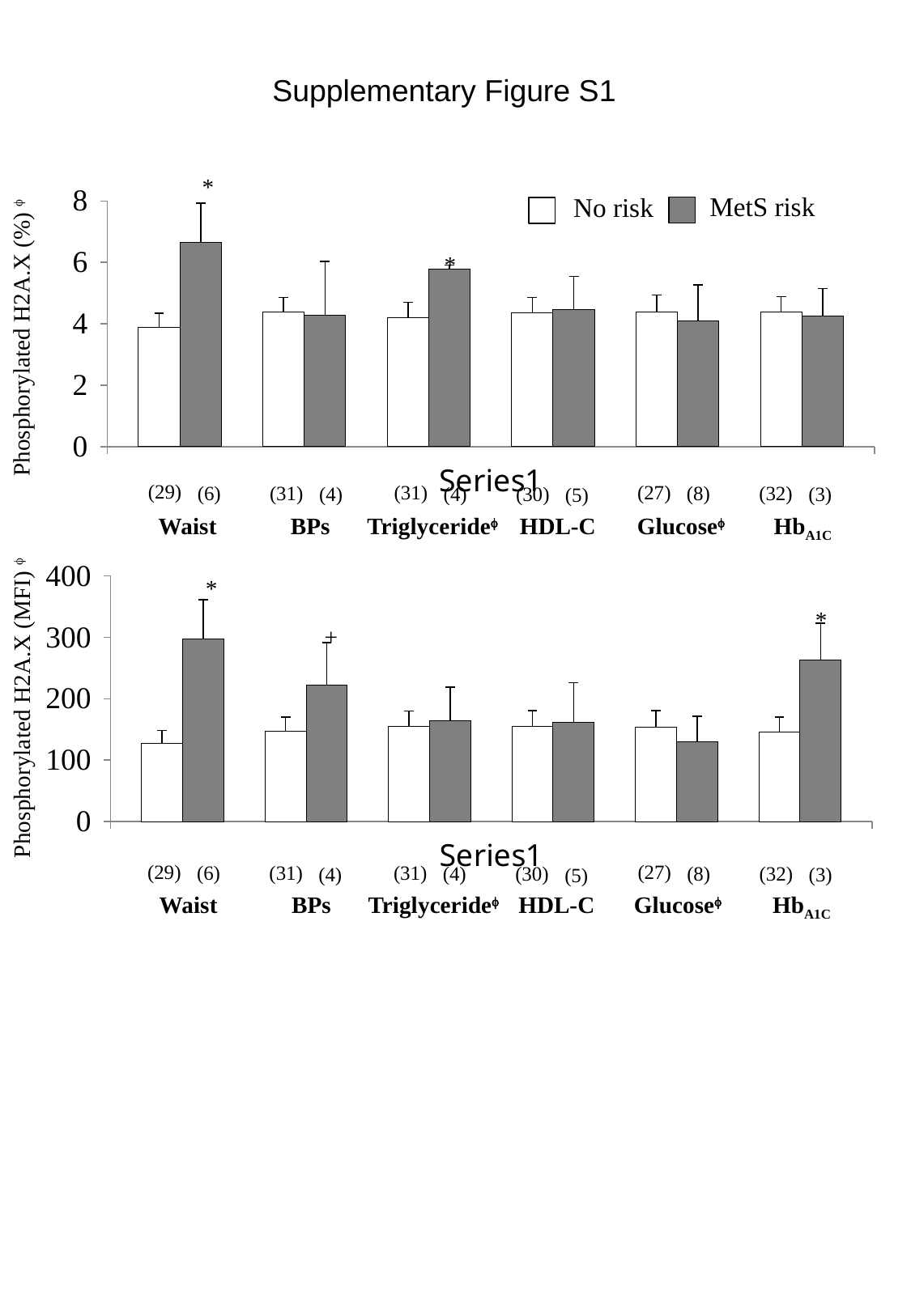

Supplementary Figure S1
*
### Chart
| Category | 계열 1 | 계열 2 | 계열 3 | 계열 4 | 계열 5 | 계열 6 | 계열 7 | 계열 8 | 계열 9 | 계열 10 | 계열 11 | 계열 12 | 계열 13 | 계열 14 | 계열 15 | 계열 16 | 계열 17 |
|---|---|---|---|---|---|---|---|---|---|---|---|---|---|---|---|---|---|
| | 3.89 | 6.65 | None | 4.38 | 4.29 | None | 4.19 | 5.77 | None | 4.35 | 4.45 | None | 4.38 | 4.09 | None | 4.38 | 4.24 |*
Phosphorylated H2A.X (%) 
(29)
(27)
(31)
(6)
(31)
(8)
(32)
(4)
(30)
(4)
(3)
(5)
Waist
BPs
Triglycerideφ
HDL-C
Glucoseφ
HbA1C
### Chart
| Category | 계열 1 | 계열 2 | 계열 3 | 계열 4 | 계열 5 | 계열 6 | 계열 7 | 계열 8 | 계열 9 | 계열 10 | 계열 11 | 계열 12 | 계열 13 | 계열 14 | 계열 15 | 계열 16 | 계열 17 |
|---|---|---|---|---|---|---|---|---|---|---|---|---|---|---|---|---|---|
| | 126.79 | 297.78 | None | 147.61 | 221.92 | None | 155.1 | 163.89 | None | 155.32 | 160.84 | None | 153.04 | 129.83 | None | 146.12 | 262.62 |*
*
+
Phosphorylated H2A.X (MFI) 
(29)
(27)
(31)
(6)
(31)
(8)
(32)
(4)
(30)
(4)
(3)
(5)
Waist
BPs
Triglycerideφ
HDL-C
Glucoseφ
HbA1C
MetS risk
No risk
